# Identification of CDKN3 as a Key Gene that Regulates Neuroblastoma Cell Differentiation

**DOI:** 10.7150/jca.89660

**Published:** 2024-01-01

**Authors:** Alexandra Vernaza, Daniela F. Cardus, Jadyn L. Smith, Veronica Partridge, Amy L. Baker, Emma G. Lewis, Angela Zhang, Zhenze Zhao, Liqin Du

**Affiliations:** Department of Chemistry and Biochemistry, Texas State University, San Marcos, Texas.

**Keywords:** cell cycle regulator, differentiation, differentiation therapy, neurite outgrowth, neuroblastoma

## Abstract

We conducted a high-content screening (HCS) in neuroblastoma BE(2)-C cells to identify cell cycle regulators that control cell differentiation using a library of siRNAs against cell cycle-regulatory genes. We discovered that knocking down expression of cyclin dependent kinase inhibitor 3 (CDKN3) showed the most potent effect in inducing neurite outgrowth, the morphological cell differentiation marker of neuroblastoma cells. We then demonstrated that CDKN3 knockdown increased expression of neuroblastoma molecular differentiation markers, neuron specific enolase (NSE), βIII-tubulin and growth associated protein 43 (GAP43). We further showed that CDKN3 knockdown reduced expression of cell proliferation markers Ki67 and proliferating cell nuclear antigen (PCNA), and reduced colony formation of neuroblastoma cells. More importantly, we observed a correlation of high tumor CDKN3 mRNA levels with poor patient survival in the investigation of public neuroblastoma patient datasets. In exploring the mechanisms that regulate CDKN3 expression, we found that multiple strong differentiation-inducing molecules, including miR-506-3p and retinoic acid, down-regulated CDKN3 expression. In addition, we found that N-Myc promoted CDKN3 expression at the transcriptional level by directly binding to the CDKN3 promoter. Furthermore, we found that CDKN3 and two additional differentiation-regulating cell cycle proteins identified in our HCS, CDC6 and CDK4, form an interactive network to promote expression of each other. In summary, we for the first time discovered the function of CDKN3 in regulating neuroblastoma cell differentiation and characterized the transcriptional regulation of CDKN3 expression by N-Myc in neuroblastoma cells. Our findings support that CDKN3 plays a role in modulating neuroblastoma cell differentiation and that overexpression of CDKN3 may contribute to neuroblastoma progression.

## Introduction

Neuroblastoma is one of the most common solid tumors of childhood 1, 2. It accounts for more than 15% of childhood cancer-related deaths 1, 3. Neuroblastoma arises from the neural crest cell precursors of the sympathetic nervous system that fail to complete the process of differentiation 4-6. For this reason, neuroblastoma cells retain some of the features of neural crest progenitor cells, including the ability to undergo neuronal differentiation upon stimulation with differentiation agents. Due to this feature, differentiation therapy, a treatment approach focusing on inducing differentiation of malignant cells, has been applied to treat neuroblastoma 2, 7. However, because the response rate of neuroblastoma patients to current differentiation agents is poor, more effective differentiation agents need to be developed for treating these resistant patients. Due to the poor understanding of the mechanisms that control neuroblastoma cell differentiation, development of new effective differentiation agents has been slow in the past few decades. Elucidating the mechanisms of cell differentiation and undercovering the key molecular pathways involved are essential for developing novel and effective approaches to differentiation therapy.

Many studies have shown that the process of cell differentiation accompanies cell cycle arrest 8-16. Cell cycle arrest has been shown to induce various cellular processes such as differentiation, senescence, and apoptosis. In neuroblastoma cells, specific cell cycle regulatory proteins have been functionally linked to cell differentiation 11, 17-20. For example, Molenaar *et al.* demonstrated that knockdown of the G_1_-entry checkpoint regulators Cyclin D1, cyclin dependent kinase 4 (CDK4) and CDK6 induced extensive neuronal differentiation, G_1_-specific cell cycle arrest, and significant cell growth arrest 21. In addition, amplification of CDK4 gene and its aberrant expression have been observed in neuroblastoma 22, 23, and increased expression of cyclin D1 has been linked to the undifferentiated phenotype of neuroblastoma 21. These studies suggest that dysregulation of cell cycle progression contributes to neuroblastoma tumorigenesis by disrupting the cell differentiation process. Targeting cancer cell cycle progression has been an important strategy for treating cancer, and inhibitors for key oncogenic cell cycle regulators have been developed to treat various types of cancers 24-26. However, the role of cell cycle progression in neuroblastoma cell differentiation process is not fully understood, which prevents the development of differentiation therapies that target cell cycle progression. For example, the role of many cell cycle regulators in neuroblastoma cell differentiation has not been investigated. A comprehensive investigation of all known cell cycle regulators may reveal the novel roles of certain cell cycle regulators in regulating neuroblastoma cell differentiation, which may provide novel targets for developing new differentiation therapy.

Using a high-content screening (HCS) approach that we previously developed 27, we systematically investigate the role of all known cell cycle regulators in modulating neuroblastoma cell differentiation by exploiting siRNAs against currently identified cell cycle-regulatory genes. Through the HCS we identified a novel mechanism centered by cyclin dependent kinase inhibitor 3 (CDKN3) that regulates neuroblastoma cell differentiation, and we further investigated the clinical relevance of this regulatory mechanism in neuroblastoma patient prognosis based on published neuroblastoma patient datasets.

## Materials and Methods

***Cell lines.*** Neuroblastoma cell lines BE^2^-C, SK-N-BE^2^ and BE^2^-M17 were from the American Type Culture Collection (ATCC), cell line LAN6 was from Children's Oncology Group (COG), and cell line KELLY was from Sigma. HEK-293T cells were from Sigma. Neuroblastoma cells were grown in DMEM/F-12 medium (Corning) supplemented with 10% Equafetal bovine serum (Atlas Biologicals). HEK-293T cells were grown in RPMI-1640 (Corning) supplemented with 5% Equafetal bovine serum.

***Reagents and materials.*** siRNAs and control oligonucleotides (oligos) were obtained from Dharmacon Inc. Transfection reagents RNAiMax were from Life Technologies. Rabbit antibodies against CDKN3, βIII-tubulin, Growth Associated Protein (GAP43), neuron specific enolase (NSE), Ki67 and Proliferating Cell Nuclear Antigen (PCNA) were from Abcam. The rabbit anti-Calnexin antibody was from Thermo Fisher. Rabbit antibodies against CDK4, CDC6 and MYCN, as well as horseradish peroxidase (HRP)-conjugated anti-rabbit IgG, were from Cell Signaling Technology.

***Detection and quantification of neurite outgrowth.*** Neuroblastoma cells were plated and treated in 96-well plates. For detecting neurite outgrowth, cells were placed into a ZOOM IncuCyte Imaging System (Essen Bioscience) and cell images were taken under 20X microscopic magnification. The neurite length associated with each treatment was calculated using the neurite definition defined specifically for each neuroblastoma cell line using the NeuroTrack system.

***Quantitative RT-PCR (qRT-PCR).*** Total RNA was isolated from neuroblastoma cells using TRIZOL (Thermo Fisher). 2 µg RNA was reverse transcribed into cDNA using a High-Capacity cDNA Reverse Transcription Kit (Applied Biosystems). An aliquot of cDNA corresponding to 50 ng of RNA was used for the qPCR amplification in an ABI 7000 using Maxima SYBR Green master mix (Thermo Fisher) with GAPDH mRNA expression as an internal control for normalization of RNA loading. Threshold cycle times (C_t_) were obtained, and relative gene expression was calculated using the comparative cycle time method.

***Western blots.*** Cell lysates were prepared using RIPA buffer. Protein concentration was determined using the Pierce BCA assay (Thermo Fisher). For electrophoresis, equal amounts of cell lysate were resolved by SDS-PAGE and transferred to PVDF membranes (Thermo Fisher). Membranes were blocked with 5% dry milk dissolved in TBST buffer, and then the membranes were probed with the primary antibodies. Bound antibodies were detected with HRP-conjugated IgG and visualized using enhanced chemiluminescent (ECL) substrate (Pierce). The images were taken by ChemiDoc XRS+ imager (Bio-Rad Laboratories). The intensities of the bands were quantified using the ImageJ software. For quantitative comparison between treatment groups, the raw band intensities in the Western blot images were quantified using ImageJ, and the relative band intensities were derived as the following: the raw band intensity of a specific protein in a specific treatment group was first normalized to its corresponding loading control (i.e., calnexin). Then the calnexin-normalized band intensity in the treatment group was further normalized to that in the control group.

***MTT assay to measure cell viability.*** Cell viability was measured by MTT (3-(4,5—Dimethylthiazol-2-yl)-2,5-Diphenyltetrazolium Bromide) assay as before 28. Briefly, cells were plated in 96-well plates and treated as specified. MTT was then added and incubated for 2 h at 37 ^o^C. Precipitates were centrifuged down and dissolved in DMSO. The absorbance at 570 nm and 630 nm were measured and the difference in the two absorbance values was calculated to determine relative cell viability.

***Colony formation assay.*** 5,000 cells were transfected with the specified oligoes at 20 nM and plated into 10 cm dishes. Two weeks later, cell colonies were stained with 0.5% crystal violet. The number and size of colonies were quantified using ImageJ 1.54d.

***Promoter luciferase reporter assay.*** The putative MYCN binding E-box was predicted using ConTra V3 prediction tool. DNA fragments of the wildtype (WT) CDKN3 promoter (positions -468 bp to -15 bp in the CDKN3 gene) containing the putative E-box binding site (CAACTG) of MYCN were amplified by PCR using Phusion enzyme (New England Biolabs) and inserted into pGL3B luciferase reporter vector (Promega) between Sac I and Hind III cloning sites to generate the WT reporter vector (P_WT_-Luc). Mutated pGL3B vector (P_MU_-Luc) containing mutant E-box site (ACATCT) was generated using the site-directed mutagenesis kit. HEK-293T cells were co-transfected with pGL3B luciferase reporter and MYCN expression vector. Luciferase activities were measured after 48 h using Luciferase Assay System (Promega) on a BioTek Synergy H4 microplate reader.

***Chromatin immunoprecipitation-PCR (CHIP-PCR) assay.*** CHIP-PCR was performed according to published procedures with some modifications 29. Briefly, four 10-cm dishes of cells were treated with 1% formaldehyde for 10 min at room temperature for cross-linking. Cross-linking was stopped by 125 mM of glycine. Cells were then washed with cold PBS, scraped into PBS containing protease inhibitors, pelleted by centrifugation, resuspended in lysis buffer (5 mM Tris, pH 8, 85 mM KCl, 0.5% NP-40, and protease inhibitors), and incubated on ice for 20 min. Cells were then ruptured by passing through a 22 gauge needle for ten times, and nuclei were pelleted, washed in lysis buffer, and resuspended in sonication buffer (10 mM Tris pH 7.5, 150 mM NaCl, 1 mM EDTA, 1% NP-40, 1% deoxycholate, 0.1% SDS, and protease inhibitors) and incubated on ice for 10 min on ice. The nuclei solution was sonicated on ice using a Tissue Tearor Homogenizer. The solution was then centrifuged, and the supernatant was pre-cleared using *bovine serum albumin (*BSA)-blocked protein A/G agarose beads for 4 h. Aliquots of the pre-cleared chromatin were then incubated with 2 µg antibody overnight at 4 ºC. As a negative control (the Input), one aliquot was incubated without antibody. Immune complexes were captured with 40 µl of pre-blocked protein A/G agarose, washed with cold washing buffer (50 mM HEPES pH 7.5, 500 mM LiCl, 1 mM EDTA, 1% NP-40, 0.7% deoxycholate and protease inhibitors) and Tris-EDTA (TE) buffer, and then resuspended in 200 µl TE. Samples were then digested with RNase A and proteinase K at 55 ºC for 3 h, then incubate at 95 ºC for 10 minutes. The co-precipitated DNA was then isolated using PCR purification column (Qiagen). The isolated DNA was eluted using 50 µl of 10 mM Tris. 1 µl of the eluted DNA was used for PCR. Primer set 1 (Forward TCCAGCGTCCCCCAAGCGCTAG, Reverse TGGGTGGCCGAAGACTCGGCC) was used to amplify the segment of the promoter containing the E-box. Primer set 2 (Forward TCTGCACCAGAGGGGAACTGTCAAA, Reverse AAGCTCTTCCATTATTTCACAGCAGCTGGC) was used to amplify a sequence located in Exon 5 as a negative control.

***Neuroblastoma patient survival analysis.*** Neuroblastoma patient analyses were based on the published neuroblastoma patient datasets in the R2 Genomics (R2) platform (http://r2.amc.nl). Three datasets were analyzed, which are the Kocak, SEQC (the seqcnb1 platform) and NRC datasets. For analyzing the survival of the whole patient population in each dataset, we included all the neuroblastoma patients who have both the tumor CDKN3 mRNA expression data and patient survival data, the two parameters required for analyzing the correlation of tumor CDKN3 mRNA level with patient survival. The patients in each dataset were separated into two groups based on the CDKN3 mRNA levels in tumor specimens, the low- and high-CDKN3 groups, by running the R2 Scan mode based on the overall patient survival data. The Scan-mode analysis sorts the tumor CDKN3 mRNA levels from low to high, and then runs every possible low- and high-group classification (i.e., for a dataset that has 283 patients, the first possible two-group classification is that the low-CDKN3 group contains 1 patient, and the high-CDKN3 group contains 282 patients, and so on) and finds the grouping that gives the maximum separation (i.e., lowest *p* value) of the overall survival curves between the low- and high-CDKN3 groups. The grouping was then applied to both overall survival and recurrence-free survival analyses, which were performed using the Kaplan-Meier method, and statistical significance between the two groups was determined by 2-tailed log-rank tests with *p* < 0.01 for both the raw and the Bonferroni adjusted *p* values considered statistically significant. To investigation the correlation between CDKN3 levels and patient overall survival in stratified subgroups, patients in the high and low CDKN3 groups are stratified by age, gender, MYCN amplification or tumor stage, and the correlation between CDKN3 levels and patient overall survival probability in each subgroup was analyzed using the Kaplan-Meier method.

***Other statistical Analysis****.* Gene expression level comparison between patient groups were performed using two-tailed Student's T-test, with *p* < 0.05 considered statistically significant. Gene expression correlation analyses were performed using Pearson correlation, with *p* < 0.05 considered statistically significant. To evaluate the effect of the siRNA library on neurite outgrowth, the *p* value for neurite length associated with each siRNA was determined by comparing it to the mean neurite length of the whole panel using multiple sample t-test. An increase in neurite length with *p* < 0.05 was considered statistically significant. For all other experiments, the statistical significance for each treatment was determined by two-tailed Student's t-test by comparing the treatment group with the control group, with *p* < 0.05 considered statistically significant.

## Results

### siRNA-based HCS in neuroblastoma cells identifies CDKN3 as the most potent cell cycle regulator that controls neurite outgrowth, a morphological differentiation marker for neuroblastoma

**Figure 1:**
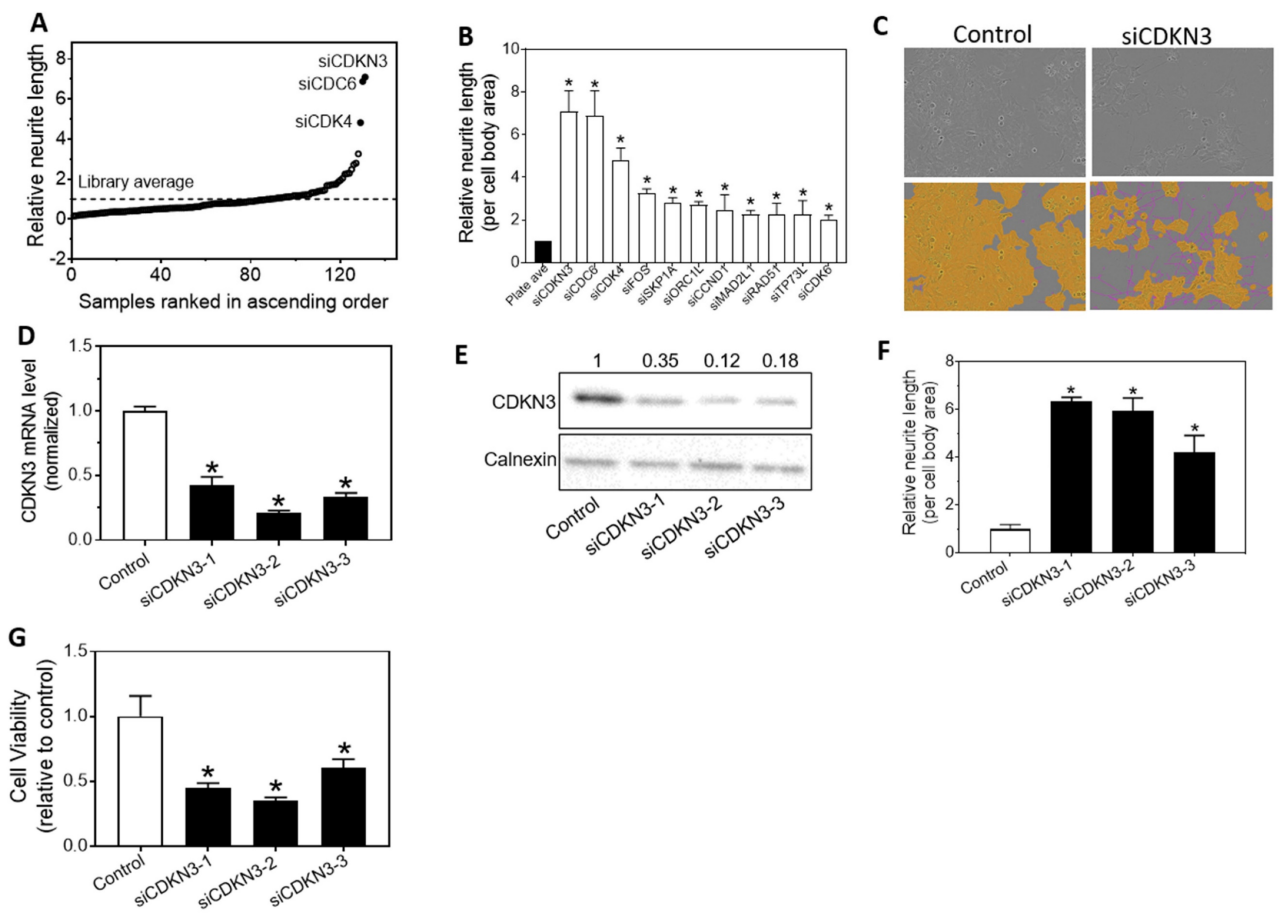
** HCS screening identifies cell cycle regulators with their knockdown inducing neurite outgrowth**. **(A-C),** BE^2^-C were transfected with 20 nM siRNAs against cell cycle regulators in 96-well plate. After 96 h transfection, cell images were taken, and neurite lengths were quantified. **A,** Scatter plot of normalized neurite lengths, sorted in ascending order, associated with individual siRNA. **B,** Neurite lengths associated with the eleven siRNAs that were identified from HCS as significantly inducing neurite outgrowth by ≥ 2 folds. *, *p* < 0.05 compared to the plate average. **C,** Representative images showing the effect of siCDKN3 on neurite outgrowth. Shown are the representative phase-contrast images, and the same images defined for cell body areas (yellow) and neurites (pink). **(D-G),** BE^2^-C were transfected with 20 nM of the siCDKN3 or negative control oligo (Control). After 96 h transfection, the effect of siCDKN3 on CDKN3 mRNA levels (**D**), CDKN3 protein levels (**E**), neurite outgrowth (**F**) and cell viability (**G**) were measured. Calnexin protein levels served as a loading control for the Western blots. The values shown above the Western blot bands are the calculated relative intensities of the corresponding bands. *, *p* < 0.05 compared to the Control oligo.

We conducted a HCS to examine the effect of depleting the expression of individual cell cycle-regulating genes on neurite outgrowth in a neuroblastoma cell line BE^2^-C, by applying a library of siRNAs against 131 genes known to regulate cell cycle progression. As shown in **Figure 1A and 1B,** we identified eleven cell cycle regulators in which expression knockdown significantly induced neurite outgrowth by ≥ 2 fold compared to the plate average, among which three genes, CDKN3, cell division cycle 6 (CDC6) and CDK4 are clearly separated from the rest of the genes in terms of their potency for inducing neurite outgrowth. The role of CDC6 and CDK4 in modulating neuroblastoma cell proliferation or differentiation has been reported previously 27, 30. We therefore focused on CDKN3 in this study. Images shown in **Figure 1C** clearly indicate siCDKN3 dramatically induces neurite outgrowth compared to cells treated with the negative control oligo.

To exclude the possibility that the siCDKN3-induced neurite outgrowth is caused by off-target effect of the synthetic siRNA oligo, we tested additional siRNA designs that target different regions of the CDKN3 mRNA. **Figure 1D-E** confirmed that all three siRNA designs successfully decreased CDKN3 expression at mRNA and protein levels. **Figure 1F** shows that the tested siRNAs dramatically induced neurite outgrowth. Correspondingly, the cell viability was also reduced by siCDKN3 (**Figure 1G**), indicating cell growth arrest accompanied neurite outgrowth induced by siCDKN3. Altogether, these results indicate the neurite outgrowth induced by siCDKN3 was not caused by off-target effect of the siRNAs.

### The differentiation-regulating functions of CDKN3 are further validated in BE^2^-C cells

**Figure 2:**
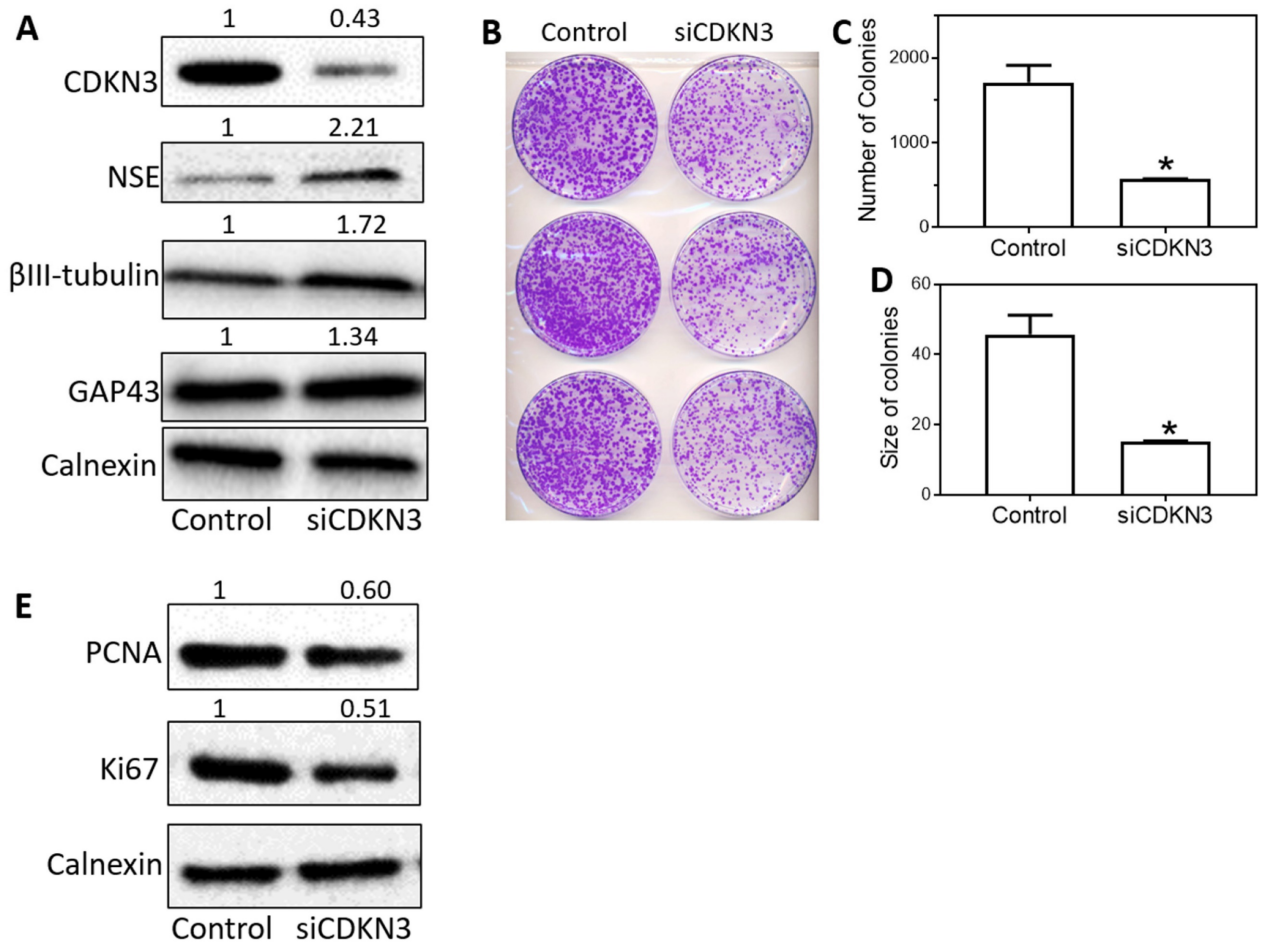
** Knockdown of CDKN3 in BE^2^-C cells increased protein expression of molecular differentiation markers and reduced colony formation of the cells. (A),** Effect of siCDKN3 on protein expressions of cell differentiation markers. BE^2^-C were transfected with 20 nM of siCDKN3 or negative control oligo (Control). After 72 h transfection, protein lysates were collected, and the protein levels of CDKN3 and molecular differentiation markers (NSE, βIII-tubulin and GAP43) were measured by Western blots, with Calnexin protein serving as a loading control. The values shown above the Western blot bands are the calculated relative intensities of the corresponding bands. **(B-D),** Effect of siCDKN3 on colony formation in BE^2^-C cells. Cells were transfected with 20 nM of siCDKN3 or negative control oligo (Control) and seeded in 100 mm dishes. Colonies were fixed and stained after 12 days, and the numbers and sizes of colonies were quantified using ImageJ. *, *p* < 0.05 compared to the Control. **B,** Effect of siCDKN3 on protein expressions of cell proliferation markers. BE^2^-C cells were transfected with siCDKN3 or control oligo as above. Protein levels of CDKN3, PCNA, Ki67 and Calnexin were measured by Western blots and quantified using ImageJ as above.

To further demonstrate that knocking down CDKN3 expression truly induces cell differentiation, we investigated the effect of CDKN3 knockdown on the expression of NSE, βIII-tubulin and GAP43 proteins, the molecular differentiation markers that have been widely used to confirm neuroblastoma cell differentiaiton 31-35. As shown in **Figure 2A**, siCDKN3 upregulated protein levels of all the three differentiation markers that have been associated with neuroblastoma cell differentiation, NSE, βIII-tubulin and GAP43 proteins. We further showed that CDKN3 knockdown decreased colony formation in BE^2^-C cells with decrements in both numbers and sizes of colonies (**Figure 2B-D**). To further confirm that the induced cell differentiation by CDKN3 knockdown is accompanied by reduced cell proliferation, we measured the protein levels of two widely used cell proliferation markers, PCNA and Ki67. As shown in **Figure 2E**, knocking down CDKN3 expression decreased protein expression of both PCNA and Ki67, further supporting that cell proliferation is truly reduced upon CDKN3 knockdown.

Together with results shown in Figure 1, our findings demonstrated that knocking down CDKN3 expression significantly induced both cell differentiation and cell growth arrest in BE^2^-C cells, suggesting the important role of CDKN3 in controlling neuroblastoma cell differentiation process.

### The differentiation-regulating functions of CDKN3 are observed in multiple neuroblastoma cell lines

**Figure 3:**
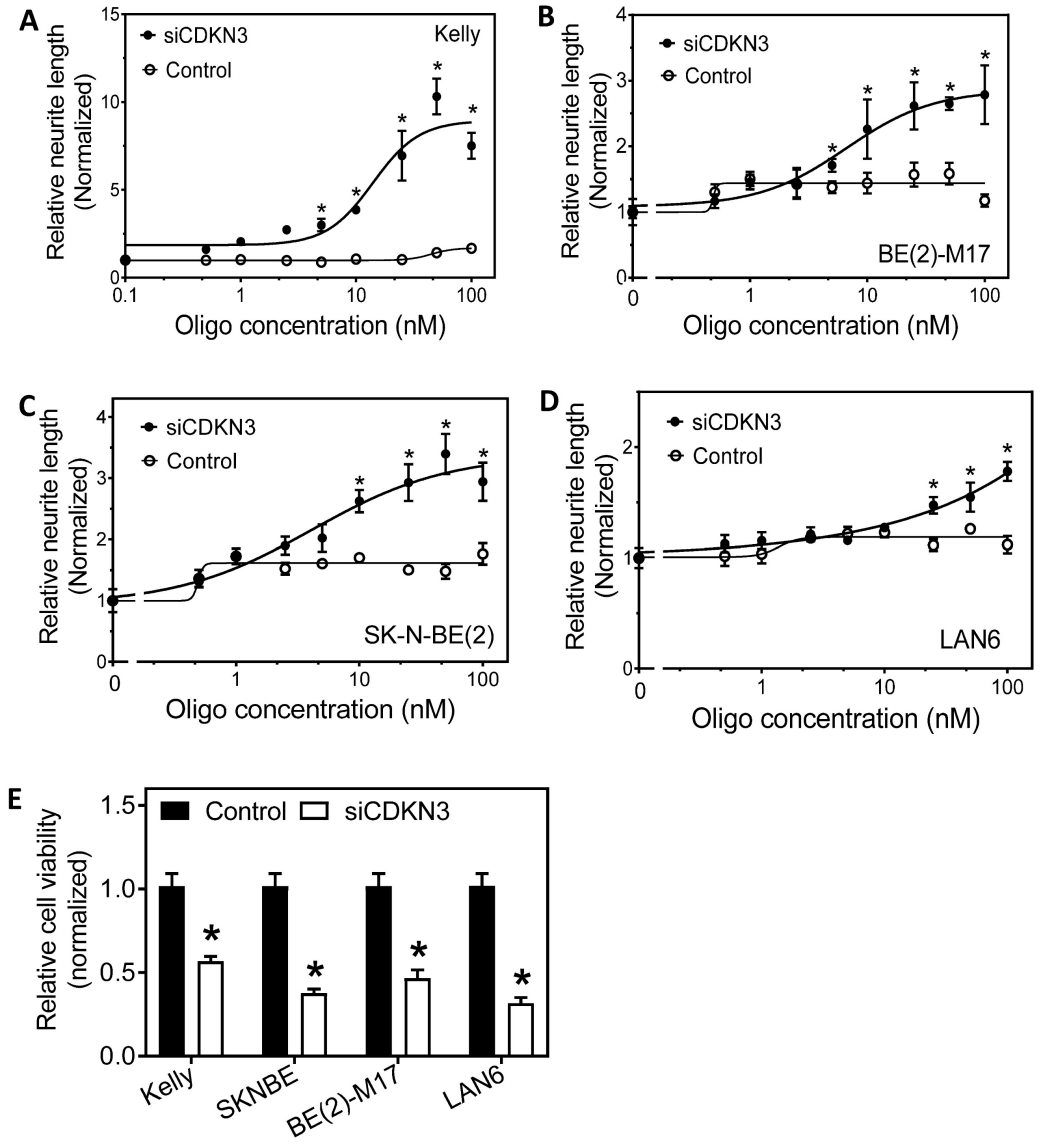
** Effect of knocking down CDKN3 expression in multiple neuroblastoma cell lines. (A-D)**, Dose-dependent effect of siCDKN3 on neurite outgrowth in Kelly, SK-N-BE^2^, BM^2^-M17, and LAN6 cells. The indicated cell lines were transfected with a serial dilution of siCDKN3 or the negative control oligo. Neurite outgrowth was measured after 96 h transfection. **E,** Effect of siCDKN3 on cell viability in the four cell lines. Cells were transfected with 20 nM of siCDKN3 for 4 days, and cell viability was measured as above. *, *p* < 0.05 compared to the Control.

To examine whether the differentiation-regulating function of CDKN3 is generic in neuroblastoma cells, we examined the effect of CDKN3 knockdown on neurite outgrowth in multiple neuroblastoma cell lines with different genetic backgrounds 36. As shown in **Figure 3A-D**, siCDKN3 induces dose-dependent neurite outgrowth in Kelly, SKNBE^2^, BE^2^-M17 and LAN6 cell lines. Correspondingly, siCDKN3 also led to a decrease in cell viability in these cell lines (**Figure 3E**). These results support the generic role of CDKN3 in modulating cell differentiation in neuroblastoma with different genetic backgrounds.

### High tumor CDKN3 mRNA levels are correlated with poor neuroblastoma patient survival

**Figure 4:**
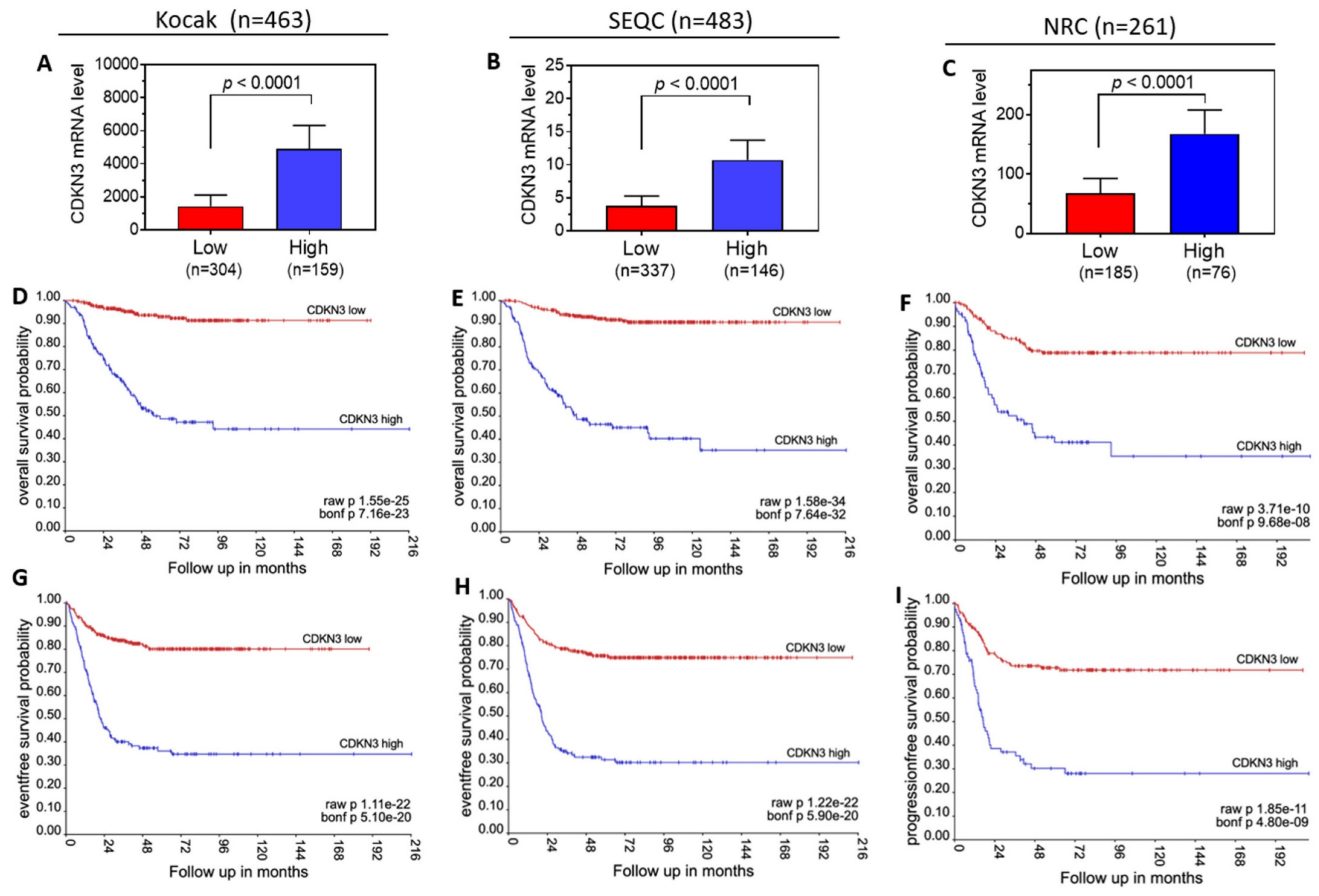
** Correlation of tumor CDKN3 mRNA levels with survival of neuroblastoma patients. (A-C),** Patients in the indicated three public neuroblastoma datasets, Kocak (**A**), SEQC (**B**), and NRC dataset (**C**) were grouped into low and high groups based on the tumor mRNA levels of CDKN3. **(D-F),** Kaplan-Meier overall survival curves generated from the Kocak (**D**), SEQC (**E**), and NRC (**F**) datasets, respectively. **(G-I),** Kaplan-Meier recurrence-free survival curves generated from the Kocak (**H**), SEQC (**H**), and NRC (**I**) datasets, respectively.

To examine the clinical relevance of elevated CDKN3 expression in neuroblastoma, we investigated the correlation of CDKN3 mRNA levels in neuroblastoma tumor specimens with patient survival based on three public patient datasets in the R2 Genomics platform. We first investigated whether CDKN3 mRNA levels are significantly correlated with patient survival in the whole patient population in each dataset. Patients in each dataset were grouped into low- and high-CDKN3 expression groups based on tumor CDKN3 mRNA levels as described in the Materials and Methods section. As shown in **Figure 4A-C,** CDKN3 mRNA levels between the high and low groups are significantly different in each dataset. Kaplan-Meier survival analyses show that patients with low tumor CDKN3 mRNA levels have both significantly higher overall (**Figure 4-F**) and recurrence-free (**Figure 4G-H**) survival probability overtime compared to patients with high CDKN3 levels in all three datasets. These results are consistent with what we observed in vitro in cell lines, with both our in vitro experimental data and patient survival analysis supporting the potential oncogenic role of CDKN3 in neuroblastoma.

Encouraged by the above finding, we further investigated the correlation between CDKN3 expression and patient survival in subgroups of neuroblastoma patients by stratifying the patients based on four clinical parameters, including age, gender, MYCN amplification status and tumor stages. As shown in **Table 1**, the correlation between high CDKN3 expression levels and poor patient survival were consistently observed in majority of the subgroups in all three datasets, including all the age and gender groups, the MYCN-nonamplified groups and the stages 2 - 4 groups, although some of the correlations did not reach statistical significance. However, correlations inconsistent with the above were observed in the MYCN-amplified group in one dataset (NRC), in the stage 1 group in one dataset (NRC), and in the stage 4s group in two datasets (Kocak and SEQC), but none of the correlations in these subgroups reached statistical significance.

### Expression levels of CDKN3 in neuroblastoma cells are down-regulated by multiple differentiation-inducing molecules

**Figure 5:**
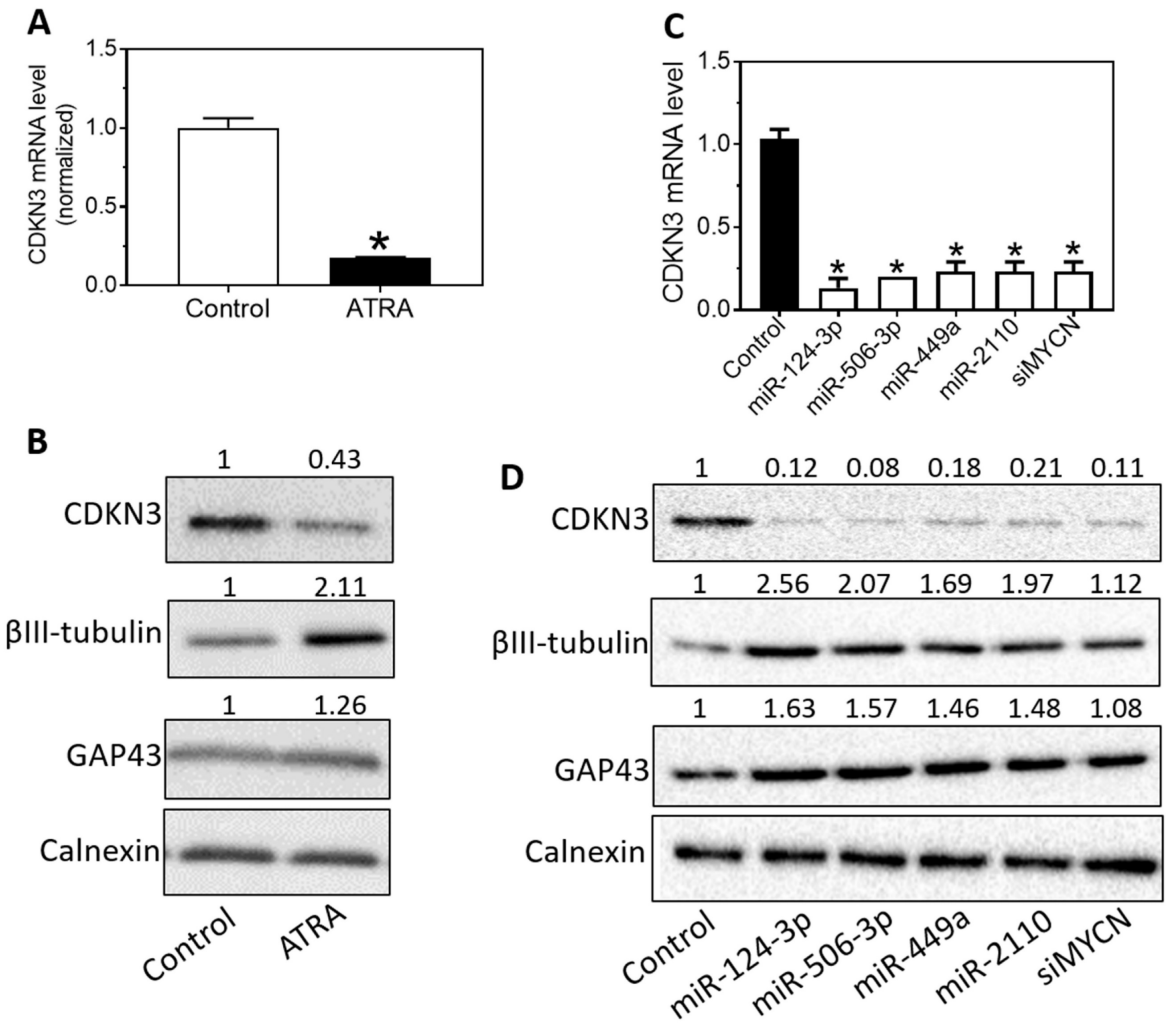
** Regulation of CDKN3 expression by differentiation-inducing molecules. (A-B),** Effect of ATRA treatment on expression of CDKN3 mRNA and protein levels. BE^2^-C cells were treated with ATRA (5 µM) for five days. RNA and protein lysates were collected and CDKN3 mRNA levels (**A**) were measured by qRT-PCR and protein levels (**B**) were measured by Western blots. (**C-D),** Effect of miRNA overexpression and MYCN knockdown on CDKN3 expression. BE^2^ cells were treated with 20 nM of miR-124-3p, miR-506-3p, miR-449a, miR-2110, siMYCN for 4 days. CDKN3 mRNA (**C**) and protein (**D**) expression were measured as above.

Many differentiation-inducing factors have been identified in neuroblastoma cells. However, the mechanisms of action of these differentiation inducers remain largely unknown. Encouraged by the above findings, we investigated the possible involvement of CDKN3 in the differentiation-inducing mechanisms of previously identified differentiation inducers, including all-trans retinoic acid (ATRA), miR-506-3p, miR-124-3p, miR-449a and miR-2110 27, 37. In addition, since MYCN is a well-known oncogene that plays a key role in maintaining the undifferentiated status of neuroblastoma cells 38, we also tested the effect of MYCN knockdown on CDKN3 expression. As shown in** Figure 5A-B,** ATRA (5 µM) dramatically decreased expression of CDKN3 expression at both the mRNA and protein levels in BE^2^-C cells. Correspondingly, ATRA increased expression of differentiation markers βIII-tubulin and GAP43, confirming that cell differentiation was truly induced by ATRA (**Figure 5B**). As shown in **Figure 5C-D**, all the four miRNA mimics dramatically decreased CDKN3 expressions at both the mRNA and protein levels. Knocking down MYCN with siRNA (siMYCN) also dramatically decreased CDKN3 mRNA and protein levels (**Figure 5D**). Correspondingly, expression of βIII-tubulin and GAP43 was also increased by the miRNA mimics and siMYCN (**Figure 5D**). Note that the extent of βIII-tubulin and GAP43 upregulation by siMYCN is not as high as that by differentiation-inducing miRNAs, which is consistent with what we have observed previously 39. These results indicate that CDKN3 expression in neuroblastoma cells is regulated by multiple differentiation-inducing mechanisms.

### Transcription of CDKN3 in neuroblastoma cells is directly regulated by MYCN

**Figure 6:**
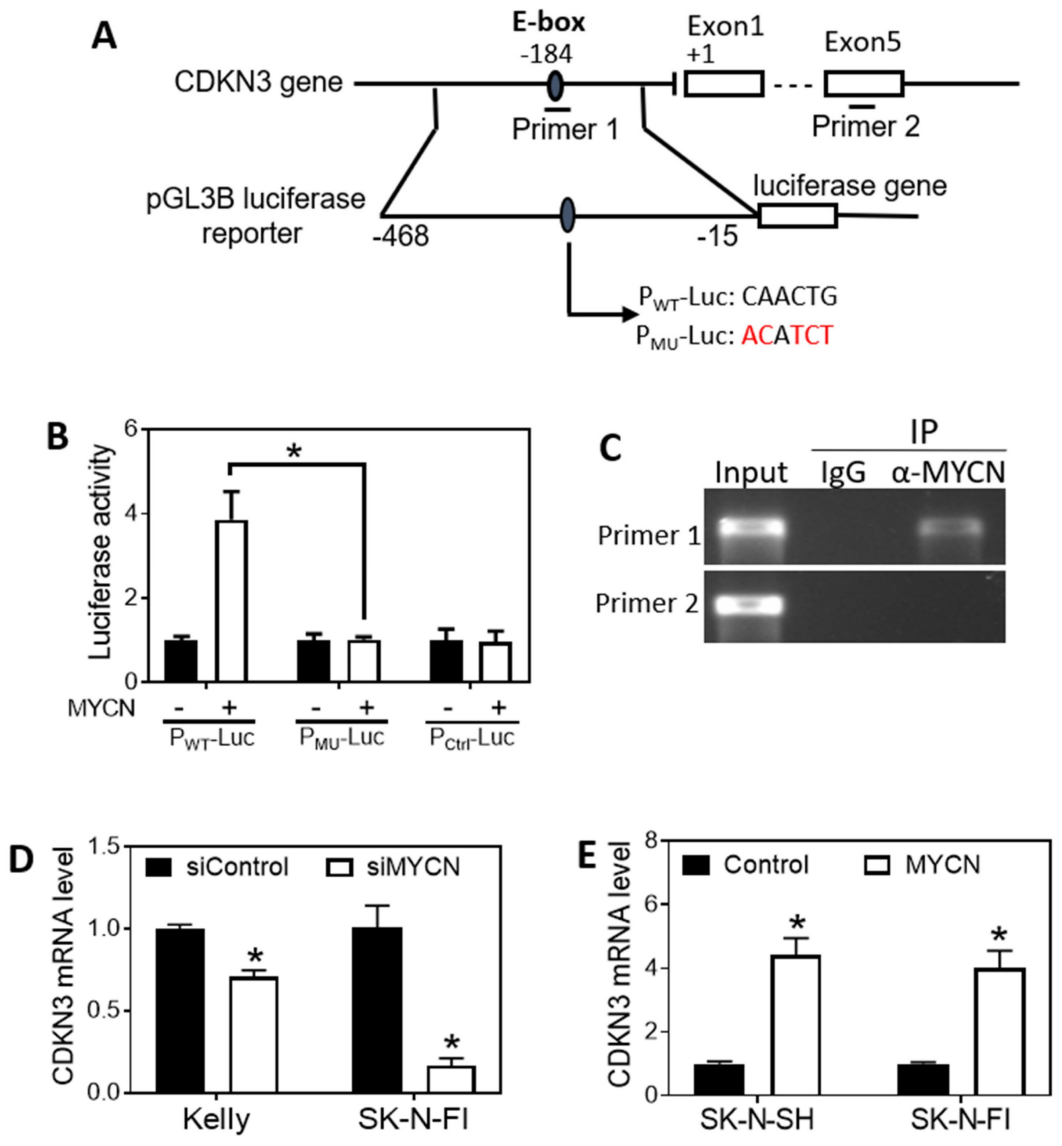
** MYCN regulates CDKN3 expression by directly binding to the E-box of the CDKN3 promoter. (A),** Schematic diagram showing the putative N-Myc binding E-box in the CDKN3 gene promoter, the amplified region for constructing the luciferase reporters, and the sites where the primer sets were designed for amplifying the DNA segment containing the E-BOX (primer 1) and for amplifying a DNA segment located within Exon 5 as a negative control of the CHIP-PCR assay (primer 2). Two luciferase reporters, P_WT_-Luc (containing the WT E-box sequence, CAACTG) and P_MU_-Luc (containing the WT E-box sequence, ACATCT) were generated. **(B),** Validation of the N-Myc binding site by luciferase assay in HEK-293T cells. A MYCN over-expression vector, MYCN (+) or a control expression vector, MYCN (-), was co-transfected with the indicated luciferase reporter or a Control reporter into cells at a concentration of 0.8 ng/μl for each vector. After 2 days, the luciferase activity was measured. *, *p* < 0.05 compared to the control vector MYCN (-).** (C),** Validation of the N-Myc binding E-box site by CHIP-PCR assay. The CHIP-PCR was performed using the indicated primers sets as illustrated in (A). IP: immunoprecipitation.** (D),** Effect of MYCN knockdown on CDKN3 mRNA expression in cell lines Kelly and SK-N-FI. Cells were transfected with 20 nM of siMYCN or negative control oligo. After 72 h, CDKN3 mRNA levels were measured as above. **(E),** Effect of MYCN overexpression on CDKN3 mRNA expression in cell lines Kelly and SK-N-FI. Cells were transfected with MYCN (+) vector or the MYCN (-) vector at a concentration of 0.8 ng/μl for each vector. After 72 h, CDKN3 mRNA levels were measured as above. *, *p* < 0.05 compared to the Control.

MYCN is a key oncogenic transcription factor that is known to regulate expression of a diverse array of genes involved in tumorigenesis 40. Since the expression of CDKN3 is down-regulated by knockdown of MYCN expression as shown above (Figure 4C-D), we investigated whether N-Myc protein directly regulates CDKN3 expression at the transcriptional level. As shown in **Figure 6A**, we found a putative N-Myc-binding E-box in the CDKN3 promoter region. We cloned a sequence of 453 base pairs (bp) in the promoter region containing the putative E-box into the upstream of the luciferase gene in the pGL3B luciferase reporter vector to construct a wild-type reporter P_WT_-Luc (**Figure 6A**). A mutant reporter (P_MU_-Luc) was generated by mutating the sequence within the E-box (**Figure 6A**). A reporter with no insertion was used as a negative control (P_ctrl_-Luc). Each reporter vector was transfected into HEK 293T cells together with a MYCN over-expression vector (MYCN (+)) or a control expression vector with no MYCN DNA insert (MYCN (-)). As shown in **Figure 6B**, MYCN overexpression significantly increased luciferase activity in P_WT_-Luc transfected cells, but not in P_MU_-Luc cells, compared to their corresponding negative controls. In addition, MYCN did not affect luciferase activity in cells transfected with the negative control reporter P_ctrl_-Luc. To further examine whether N-Myc protein directly binds to the E-box, we performed CHIP-PCR in BE^2^-C cells. **Figure 6A** illustrates the positions of the DNA sequences to be amplified by each PCR primer set, with primer 1 used to amplify the sequence containing the predicted N-Myc-binding E-box and primer 2 used to amplify a sequence located in Exon 5 as a negative control. As shown in **Figure 6C**, primer 1, but not primer 2, successfully amplified its targeted sequence, indicating anti-N-Myc antibody (α-N-Myc) successfully pulled down the predicted N-Myc-binding sequence but not the control sequence. In addition, non-specific IgG did not pull down any of the above sequences, excluding the possibility of non-specific pulldown of protein-DNA complexes by IgG. Together, these results demonstrate that N-Myc activates CDKN3 transcription through directly binding to the identified N-Myc-binding E-box in the promoter region of CDKN3 gene.

We further examined the effect of N-Myc on CDKN3 mRNA levels in additional neuroblastoma cell lines. As shown in **Figure 6D**, knockdown of MYCN expression resulted in a significant decrease of CDKN3 mRNA levels in two MYCN-amplified cell lines Kelly (with highly expressed MYCN mRNA level) and SK-N-FI (with moderately expressed MYCN mRNA level). In contrast, over-expression of MYCN increased CDKN3 mRNA levels in SK-N-FI and SK-N-SH (MYCN-non-amplified) cells (**Figure 6E**). These results further support that the transcriptional regulation of the CDKN3 gene by N-Myc is a generic mechanism in neuroblastoma cells with different genetic backgrounds.

### Expression levels of CDKN3 positively correlate with MYCN expression in neuroblastoma tumor specimens

**Figure 7:**
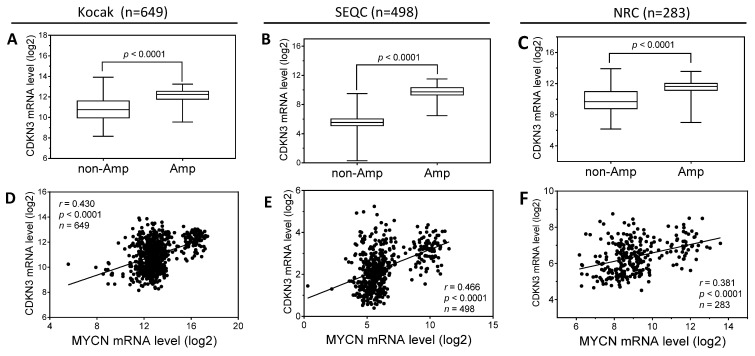
** The correlation of CDKN3 and MYCN expression in neuroblastoma tumor specimens. (A-C),** Comparison of CDKN3 mRNA levels between MYCN amplified and non-amplified neuroblastoma tumors from the indicated three patient datasets. **(D-F),** The correlation of tumor CDKN3 and MYCN mRNA expression levels in the indicated three patient datasets.

To examine the clinical relevance regarding the transcriptional regulation of CDKN3 expression by N-Myc, we analyzed the above three patient datasets for the correlation between MYCN and CDKN3 mRNA levels. As shown in **Figure 7A-C**, the CDKN3 mRNA levels in the MYCN-amplified tumors are significantly higher than those in MYCN-nonamplified tumors in all the three datasets. In addition, the expression of CDKN3 mRNA levels is significantly positively correlated with MYCN mRNA levels in all three datasets (**Figure 7D-F**).

Coupled with our findings that CDKN3 knockdown induced cell differentiation and cell growth arrest as shown above, these results altogether suggest that CDKN3 represents one of the molecular pathways that mediate the oncogenic function of MYCN.

### CDKN3, CDC6 and CDK4 reciprocally modulate expression of each other

**Figure 8:**
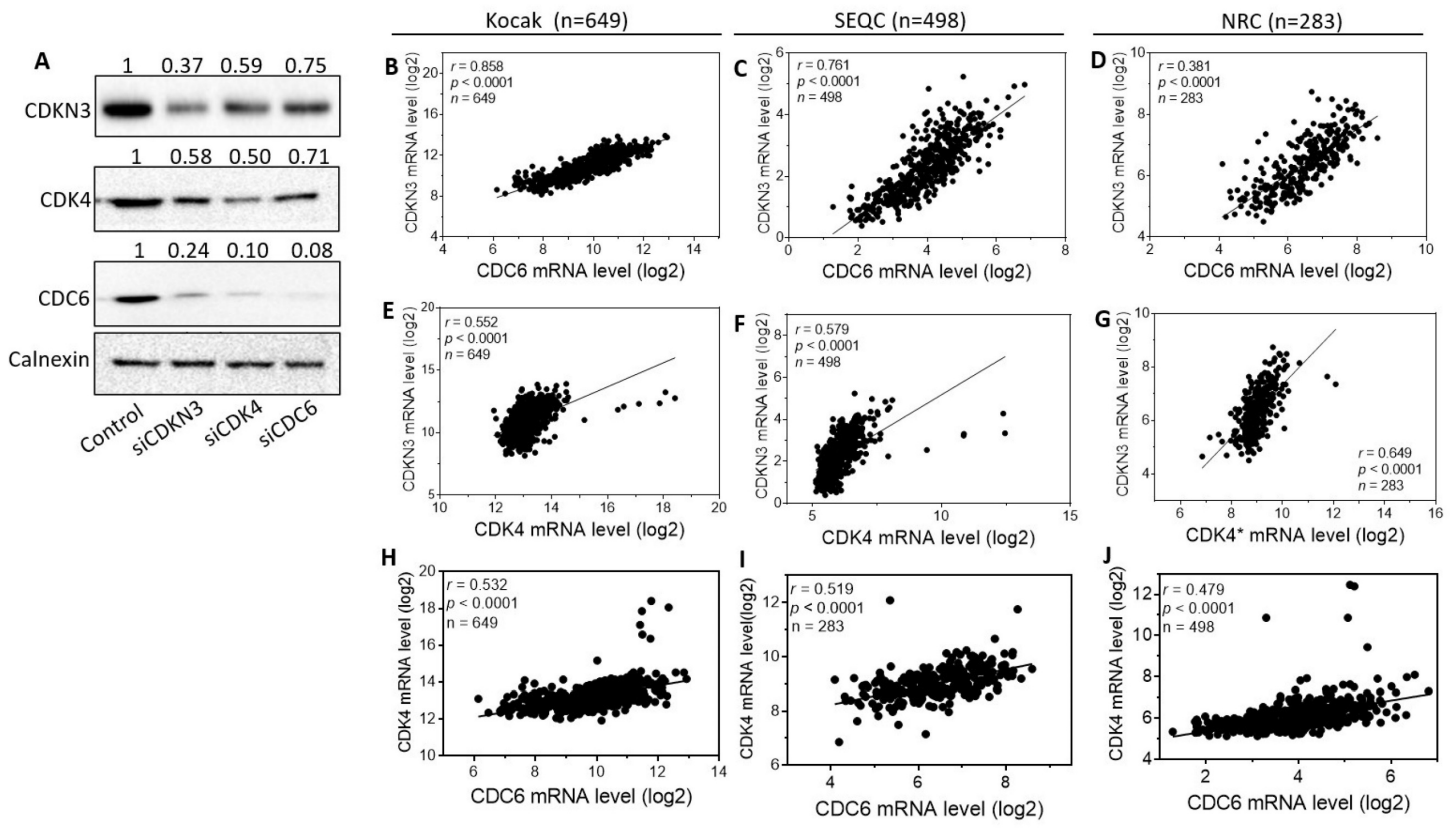
** The reciprocal regulations of expression between CDKN3, and CDC6 and CDK4 in neuroblastoma cell lines, and the correlations of their mRNA levels with each other in neuroblastoma tumor specimens. (A),** Effect of knocking down expression of CDKN3, CDK4 and CDC6 on the protein levels of CDKN3, CDK4 and CDC6, respectively. Cells were transfected with 20 nM of the indicated oligos. After 72 h, protein levels of CDKN3, CDK4 and CDC6 were detected by Western blots. **(B-D),** Correlation of CDKN3 mRNA levels with CDC6 mRNA levels in the three public patient datasets. **(E-G),** Correlation of CDKN3 mRNA levels with CDK4 mRNA levels in the indicated datasets. **(H-J),** Correlation of CDK4 mRNA levels with CDC6 mRNA levels in the indicated patient datasets. CDK4*, the gene symbol in the NRC dataset is CDK4, TSPAN31.

**Figure 9:**
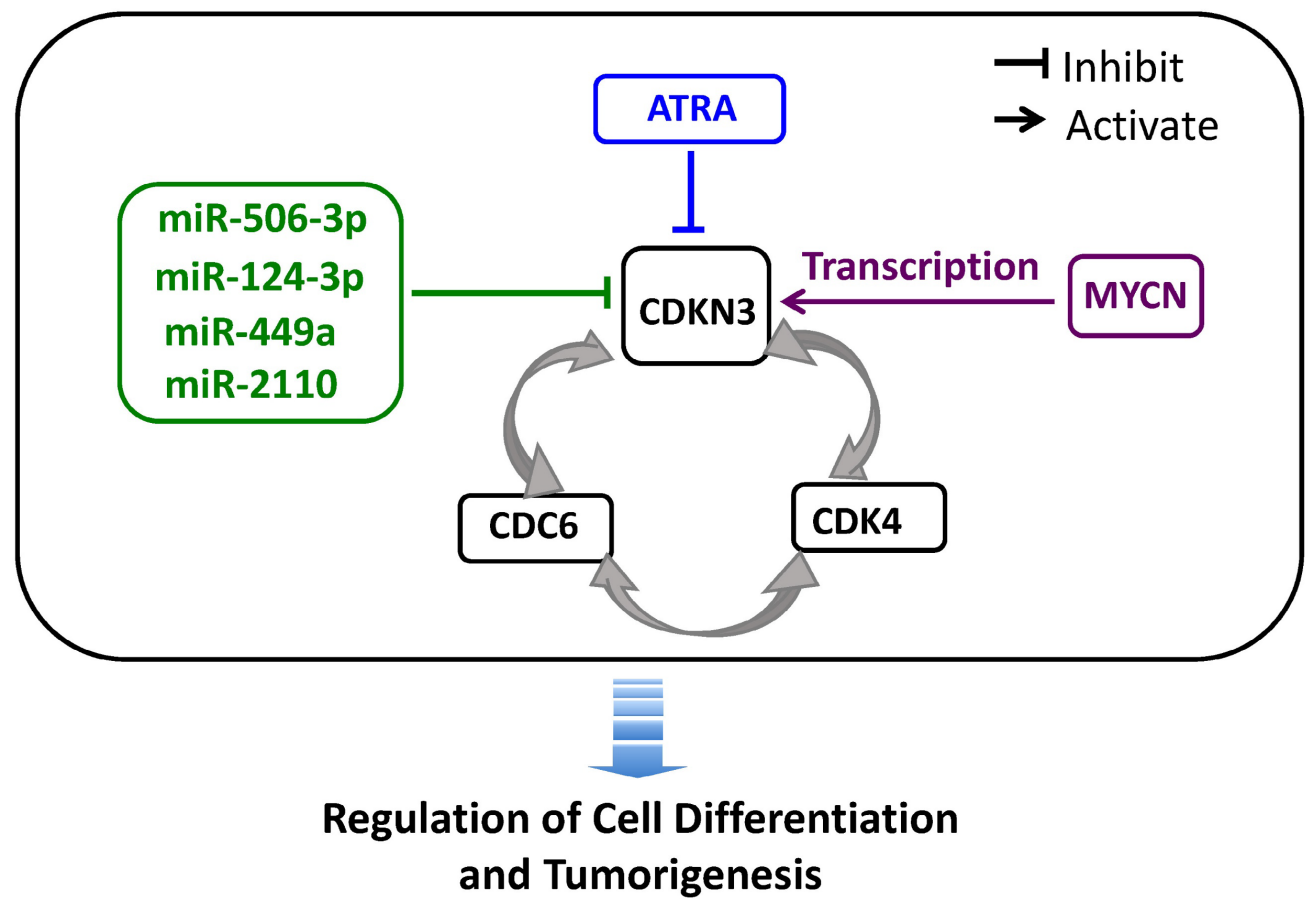
** Schematic diagram showing the interplay of CDKN3 with other differentiation-regulating molecules.** The diagram summarizes our findings regarding the interaction of CDKN3 with other differentiation-regulating molecules: 1 the differentiation-inducing molecules ATRA, miR-506-3p, miR-124-3p, miR-449a and miR-2110 function as repressor of CDKN expression, 2 N-Myc transcriptionally activates CDKN3 gene expression, and 3 CDKN3, CDC6 and CDK4 form a positive regulatory loop to up-regulate expression of each other.

As shown in Figure 1A-B, our HCS identified two additional cell cycle regulators that have strong differentiation-regulating functions, CDC6 and CDK4. We intended to understand the relationship of these three differentiation-regulating cell cycle proteins in neuroblastoma cells. We investigated whether they reciprocally regulate expression of each other. As shown in **Figure 8A**, knockdown of CDKN3, CDC6 and CDK4 individually decreased expression of each other. These results reveal an interesting regulatory network formed by the three cell cycle regulators (**Figure 9**). To examine the clinical relevance of this reciprocal regulatory network in neuroblastoma patients, we examined the correlations between the CDKN3, CDK4 and CDC6 mRNA levels in the above three patient datasets. As shown in **Figure 8B-G**, CDKN3 mRNA levels are significantly positively correlated with expression levels of both CDC6 (**Figure 8B-D**) and CDK4 (**Figure 8E-G**) in all the three datasets. Likewise, CDC6 and CDK4 mRNA levels are also significantly correlated (**Figure 8H-J**). Altogether, these results support the clinical significance of the interaction network formed by CDKN3, CDC6 and CDK4 in neuroblastoma.

## Discussion

Through a HCS screening, we identified CDKN3 as a strong regulator of neuroblastoma cell differentiation. In addition, we preliminarily characterized the interaction of CDKN3 with several key differentiation-regulating molecules (Figure 9). The results suggest that CDKN3 plays an important role in the cell signaling pathways that determine neuroblastoma cell fate, with depletion of CDKN3 expression promoting cell differentiation. The novelty of our study lies in three aspects: 1) We are the first to characterize the function of CDKN3 in modulating neuroblastoma cell differentiation. Previous studies have characterized the function of CDKN3 in modulating cell survival and proliferation in multiple cancer types 41. However, no published study has been conducted to systematically characterize the role of CDKN3 in regulating cell differentiation in neuroblastoma. 2) We utilized a novel functional high-content screening approach to identify the differentiation-modulating function of CDKN3 in neuroblastoma cells, which is different from the approaches used in all previous CDKN3 studies. One advantage of our functional screening approach is that it allows a direct and systematic comparison of all the screened cell cycle regulators. Our results show that knocking down CDKN3 expression has the strongest differentiation-inducing effect among the tested cell cycle regulators. 3) We for the first time characterized CDKN3 gene as a transcriptional regulatory target of N-Myc, and we demonstrated that N-Myc promotes CDKN3 transcription through a N-Myc binding site located upstream of the CDKN3 protein coding region.

Cyclin dependent kinase inhibitors (CDKNs, CDKIs) are a group of proteins that play important roles in modulating CDK activities by dephosphorylating CDKs 42. In addition to regulating cell cycle progression 42, CDKNs have been found to regulate other important cellular processes, such as apoptosis, cell fate determination and cell migration 43. Multiple CDKNs have been demonstrated to play important roles in tumorigenesis. For example, CDKN1B, also called p27 or p27/KiP1, was found to promote the function of the tumor suppressor Phosphatase and Tensin Homolog (PTEN) by stabilizing PTEN in the cytoplasm, functioning as a tumor suppressor 44. Although the molecular mechanisms by most of the CDKNs regulating the tumorigenesis process are far from fully elucidated, findings from published studies have clearly supported that different CDKNs have distinct roles in the tumorigenesis processes due to the unique interactive network of each CDKN formed with CDKs or other protein partners. Some CDKNs have been investigated in neuroblastoma cells. For example, overexpression of CDKN1B in mouse neuroblastoma cells was found to induce cell differentiation 45. However, the role of CDKN3 in modulating neuroblastoma cell differentiation has not been investigated previously. CDKN3 is a relatively unique member of the CDKN family because overexpression of CDKN3 has been proven to be oncogenic in multiple cancer types 41, unlike the tumor suppressive CDKNs, such as CDKN1A and CDKN1B. For example, in a study conducted in ovarian cancer, CDKN3 protein was found to be overexpressed by 3.35-fold in epithelial ovarian cancer specimens compared to healthy ovarian epithelium, and this overexpression was correlated with lower patient survival 46. The authors further showed that inhibition of CDKN3 expression in an ovarian cancer cell line OVCAR3 inhibited cell growth and proliferation 46. In a study conducted in gastric cancer, 35 of the 90 patients had significantly elevated expression of CDKN3 compared to normal adjacent tissues 47. Longitudinal study of the 90 patients over a period of 5 years showed a higher survival rate in patients who had lower CDKN3 mRNA expression 47. The authors further showed that knockdown of CDKN3 expression resulted in reduced cancer metastasis and adhesion 47. In a more recent study, CDKN3 was found to be significantly upregulated in the GC group than in the control group, consistent with the above findings 48. The oncogenic function of CDKN3 was also reported in esophageal squamous cell carcinoma (ESCC) tissues 49. This study showed that the expression of CDKN3 was significantly upregulated in ESCC tissues compared to normal tissues 49. Their functional assays further revealed that CDKN3 knockdown decreased the ability of ESCC cells to proliferate, invade and migrate 49. CDKN3 has also been investigated in cancers of neuronal origin. For example, in a study conducted in Glioblastoma multiforme (GBM), it was shown that CDKN3 protein was downregulated in GBM compared to normal tissue and CDKN3 played a role in controlling mitosis 50. A study conducted in neuroblastoma showed that the promoter of the CDKN3 gene was hypomethylated in neuroblastoma cells and this change was associated with increased expression of CDKN3 51. However, the role of CDKN3 in regulating neuroblastoma cell differentiation has not been reported previously. Our results presented in this study for the first time demonstrate that CDKN3 plays an important role in regulating neuroblastoma cell differentiation.

The mechanisms of regulating CDKN3 expression have been investigated in previous studies. A study showed that miR-181d-5p directly repressed CDKN3 protein expression by binding to a target site in the 3'UTR of CDKN3 mRNA in non-small cell lung cancer 52, and this regulation was later confirmed by another research group 53. Another study showed that a transcriptional regulator Yin Yang-1 (YY1) downregulated the expression of CDKN3 by directly binding to the promoter region of CDKN3 in pancreatic cancer cells 54. In our current study, we discovered that several differentiation-inducing molecules, including four differentiation-inducing miRNAs (miR-506-3p, miE-124-3p, miR-449a and miR-2110) and retinoic acid inhibited CDNK3 expression in neuroblastoma cells. However, informatics analysis showed that the 3'UTR of CDKN3 mRNA does not have target sites of the four miRNAs. Also, we did not identify any binding sites for retinoic acid receptor (RAR) and retinoid X receptor (RXR), which are transcription factors that bind and are activated by retinoic acids, in the promoter region of CDKN3 genes. These findings indicate that these miRNAs and retinoic acids regulate CDKN3 expression through indirect signaling pathways. These pathways certainly need to be elucidated in future studies.

Another finding in our study is that MYCN directly regulates CDKN3 expression at the transcription level. More importantly, we found that MYCN mRNA levels in neuroblastoma tumor specimens are positively and significantly correlated with CDKN3 mRNA levels in all the three independent patient datasets. In addition, we found that CDKN3 mRNA levels are significantly higher in MYCN-amplified tumors than in MYCN non-amplified tumors in all three patient datasets. These results strongly support that N-Myc plays a key role in controlling CDKN3 expression. Together with the oncogenic function of CDKN3 that we observed as above, these results suggest that CDKN3 may represent one of the key signaling pathways underlying the congenic function of N-Myc in neuroblastoma.

Another interesting finding in our study is that the top three differentiation-regulating cell cycle regulators identified in our HCS, CDKN3, CDC6 and CDK4, form a reciprocal interaction network to promote expression of each other. More importantly, we found that the expression levels of these three genes are significantly correlated with each other in neuroblastoma patients. These findings suggest the importance of this inter-regulative network in controlling neuroblastoma cell differentiation. However, there are many questions that need to be answered regarding this interaction network. For example, would overexpression of one of the proteins diminish or abolish the differentiation-inducing activity of knocking down one of the other two? Are there physical interactions between these three proteins in their cell differentiation-regulating mechanisms? We expect that these questions will be answered in future studies.

To preliminarily evaluate the potential clinical relevance of our laboratory findings in neuroblastoma patients, we investigated the correlation of neuroblastoma tumor CDKN3 mRNA expression with patient survival. We observed a correlation between high CDKN3 mRNA levels and poor patient survival in three independent patient populations. After stratifying the patients by age, gender, MYCN amplification status and tumor stage, the correlations between high CDKN3 expression and poor patient survival were consistently observed in most of the stratified subgroups, although some of the correlations did not reach statistical significance; the statistical insignificance in some subgroups is most likely due to the decreased sample sizes when the patients are divided into subgroups. However, inconsistency was observed in the MYCN-amplified group, the stage 1 group and the stage 4s group. Specifically, in the MYCN-amplified group of the NRC dataset, the high CDKN3 patients exhibited a slightly higher survival probability than the low CDKN3 group (0.24 *vs* 0.2), but the *p* values did not reach statistical significance. Given the inconsistency observed in the MYCN-amplified group in the three datasets, the relationship between CDKN3 expression and patient survival in MYCN-amplified patients cannot be concluded based on our current study. Since MYCN-amplification is such a strong driving force of poor prognosis, the effect of any additional oncogenic factor can only be observed when the sample size is large enough. Therefore, the contribution of CDKN3 overexpression to prognosis in MYCN-amplified patients should be further evaluated in much larger patient populations in the future. Another inconsistency was observed in stage 1 patients, with both the low and high CDKN3 groups having a survival probability of 1 in the NRC dataset. This is not surprising given the well-known good prognosis of stage 1 patients and the small samples size of stage 1 patients in the NRC dataset. Given the statistical significance observed in the other two datasets for stage 1 patients, our results support the correlation of high CDKN3 level with low patient survival in stage 1 patients, although this will need to be further validated in future studies in additional neuroblastoma patients. Inconsistency was also observed in stage 4s patients, with none of the correlations in the three datasets reaching statistical significance. Stage 4s neuroblastoma was defined as a 'special' stage of disseminated neuroblastoma that has a good prognosis 55, 56. Given the narrow differences of survival probabilities among the Stage 4s subgroups shown in all the three datasets (0.84 - 1), we speculate that statically distinguishing the survival probabilities between high and low CDKN3 groups in Stage 4s patients would require much larger sample sizes. In conclusion, by stratifying the patients based on age, gender, MYCN amplification status and tumor stage, results from the three datasets clearly supported the correlation of high CDKN3 level with poor patient survival probability in all age and gender groups, in MYCN-nonamplified group and in stages 2 - 4 groups. The correlations between CDKN3 expression and survival in the MYCN-amplified, stage 1 and stage 4s patients, however, need to be further investigated in patient datasets with larger sample sizes in the future. In addition, our current clinical investigations were retrospective studies based on pre-existing publicly available datasets. To fully characterize the clinical significance of CDKN3 in predicting patient prognosis, future prospective studies based on newly recruited neuroblastoma patients are needed.

## 5. Conclusions

We identified CDKN3 as potent modulator of neuroblastoma cell differentiation. In addition, we found that the expression of CDKN3 is regulated by multiple key differentiation-regulating molecules, including four differentiation-inducing miRNAs, retinoic acid and N-Myc, suggesting that CDKN3 may represents a key signaling pathway underlying the differentiation-regulating function of these molecules. Furthermore, we found that CDKN3 modulates expression of the other two differentiation-regulating cell cycle regulatory proteins, CDC6 and CDK4. These results altogether suggest that multiple differentiation-regulating pathways converge at CDKN3 and targeting CDKN3 expression may be an effective approach for neuroblastoma differentiation therapy. Many questions regarding the mechanisms underlying this regulatory network need to be answered in future studies, which are imperative for eventually translating the knowledge on this interaction network into neuroblastoma treatment. Since the main goal of the current study is to experimentally characterize the function of CDKN3 in cultured neuroblastoma cells, we only preliminarily investigated the potential clinical relevance of our laboratory findings. Our investigation based on public neuroblastoma datasets preliminarily supports the correlation of high tumor CDKN3 mRNA levels with poor patient survival in most of the patient subgroups. Future prospective studies with larger sample sizes are certainly needed to further validate the clinical significance of CDKN3 overexpression in neuroblastoma.

### Funding

This work was partially supported by the Academic Research Enhancement Award (AREA) grant (R15CA249653-01) from the National Cancer Institute, National Institute of Health (to L.D.) and South Texas Doctoral Bridge Program (R25 Bridges to the Doctorate Program GM102783) from National Institute of General Medical Sciences, National Institute of Health (to A.V. and D.F.C.).

### Author Contributions

V.P., A.L.B. and Z.Z. performed the experiments. A.V., J.L.S., D.F.C., E. G.L. and A.Z. performed data analysis. A.V. played the major role in leading the data analysis and in revising the manuscript. L.D. designed the study and wrote the manuscript with the help of all other authors.

## Figures and Tables

**Table 1:** ** Correlations between CDKN3 levels and patient survival in stratified patient groups in three patient datasets.** Shown in the columns are the clinical parameter, subgroup, number of patients in the high and low CDKN3 groups, average CDKN3 mRNA level in high and low CDKN3 groups, and survival probability and *p* value in the high and low CDKN3 groups.

**Kocak Dataset**													
**Parameter**	**Subgroup**	** Number of patients (n)**				**Average CDKN3 mRNA level**				**Survival probability**			
		**Total**	**High CDKN3 Group**	**Low CDKN3 Group**		**Total**	**High CDKN3 group**	**Low CDKN3 group**		**High CDKN3 group**	**Low CDKN3 group**	**Raw *p* value**	**Bonf *p* value**
**All**	**N/A**	**463**	**159**	**304**		**2613.16**	**4884.07**	**1425.41**		**0.44**	**0.91**	**1.55E-25**	**7.16E-23**
													
**Age**	< 18 mos	302	73	229		2216.59	4595.04	1458.40		0.67	0.98	1.71E-14	5.04E-12
	> 18 mos	161	86	75		3357.02	5129.42	1324.68		0.23	0.78	3.35E-08	5.09E-06
													
**Sex**	Male	257	88	169		2670.90	4987.40	1464.68		0.44	0.85	1.43E-09	3.56E-07
	Female	191	68	123		2604.83	4786.84	1398.52		0.32	0.96	2.50E-20	4.54E-18
													
**MYCN**	Amp	65	55	10		4726.23	5212.31	2052.79		0.25	0.28	0.218	1
	Non-amp	393	102	291		2263.83	4716.80	1404.03		0.53	0.94	4.38E-18	1.71E-15
													
**Tumor Stage**	1	113	11	102		1553.19	4282.31	1258.87		0.71	1	6.29E-08	6.48E-06
	2	77	12	65		1771.91	3691.98	1417.44		0.55	0.98	3.31E-04	0.021
	3	71	21	50		2666.54	5176.17	1612.50		0.28	0.92	1.23E-08	6.90E-07
	4	142	95	47		3896.80	5039.47	1587.15		0.33	0.78	7.60E-05	0.01
	4s	60	20	40		2587.89	4885.46	1439.10		0.98	0.84	0.045	1
**SEQC dataset**													
**Parameter**	**Subgroup**	** Number of patients (n)**				**Average CDKN3 mRNA level**				**Survival probability**			
		**Total**	**High CDKN3 group**	**Low CDKN3 group**		**Total**	**High CDKN3 group**	**Low CDKN3 group**		**High CDKN3 group**	**Low CDKN3 group**	**Raw *p* value**	**Bonf *p* value**
**All**	**N/A**	**483**	**146**	**337**		**5.91**	**10.75**	**3.82**		**0.35**	**0.91**	**1.58E-34**	**7.64E-32**
													
**Age**	< 18 mos	298	55	243		4.95	10.06	3.79		0.71	0.97	5.98E-14	1.73E-11
	> 18 mos	185	91	94		7.47	11.16	3.89		0.23	0.93	3.77E-11	6.72E-09
													
**Sex**	Male	277	84	193		6.00	10.75	3.93		0.45	0.92	2.32E-18	6.32E-16
	Female	206	62	144		5.80	10.74	3.68		0.15	0.89	8.23E-21	1.61E-18
													
**MyCN**	Amp	91	69	22		9.49	10.85	5.22		0.29	0.39	0.125	1
	Non-amp	387	76	311		5.08	10.64	3.72		0.4	0.94	4.79E-26	1.85E-24
													
**Tumor stage**	1	117	7	110		3.61	8.35	3.30		0.88	1	1.83E-04	0.019
	2	77	7	70		4.17	8.85	3.70		0.72	0.98	1.50E-03	0.094
	3	63	17	46		5.77	11.05	3.82		0	0.85	1.86E-15	8.92E-14
	4	174	106	68		8.54	11.00	4.71		0.22	0.73	7.16E-07	1.20E-04
	4s	52	9	43		5.06	10.49	3.93		1	0.86	0.15	1
**NRC dataset**													
**Parameter**	**Subgroup**	** Number of patients (n)**				**Average CDKN3 mRNA level**				**Survival probability**			
		**Total**	**High CDKN3 group**	**Low CDKN3 group**		**Total**	**High CDKN3 group**	**Low CDKN3 group**		**High CDKN3 group**	**Low CDKN3 group**	**Raw *p* value**	**Bonf *p* value**
**All**	**N/A**	**261**	**76**	**185**		**96.83**	**167.88**	**67.65**		**0.35**	**0.79**	**3.71E-10**	**9.68E-08**
													
**Age**	< 18 mos	136	27	109		83.95	158.48	65.49		0.54	0.94	5.66E-07	7.31E-05
	> 18 mos	125	49	76		110.85	173.06	70.74		0.18	0.78	3.34E-05	3.91E-03
													
Sex*	Male	N/A	N/A	N/A		N/A	N/A	N/A		N/A	N/A	N/A	N/A
	Female	N/A	N/A	N/A		N/A	N/A	N/A		N/A	N/A	N/A	N/A
													
**MYCN**	Amp	53	29	24		135.10	174.41	87.59		0.24	0.2	0.078	1
	Non-amp	205	46	159		87.04	164.18	64.72		0.37	0.87	3.86E-08	7.84E-06
													
**Tumor stage**	1	45	4	41		68.67	141.98	61.52		1	1	1	1
	2	33	6	27		79.26	143.47	65.00		0.83	0.96	0.307	1
	3	41	14	27		109.13	192.71	65.79		0.55	0.87	0.071	1
	4	117	48	69		112.58	166.11	75.33		0.1	0.49	2.85E-04	0.031
	4s	24	4	20		78.47	164.68	61.23		0.86	1	0.119	1

*, Sex is not identified in the NRC dataset.

## References

[1] Maris JM, Hogarty MD, Bagatell R, Cohn SL (2007). Neuroblastoma. Lancet.

[2] Park JR, Eggert A, Caron H (2010). Neuroblastoma: biology, prognosis, and treatment. Hematol Oncol Clin North Am.

[3] Buechner J, Einvik C (2012). N-myc and noncoding RNAs in neuroblastoma. Mol Cancer Res.

[4] Hoehner JC, Gestblom C, Hedborg F, Sandstedt B, Olsen L, Pahlman S (1996). A developmental model of neuroblastoma: differentiating stroma-poor tumors' progress along an extra-adrenal chromaffin lineage. Laboratory investigation; a journal of technical methods and pathology.

[5] Edsjo A, Holmquist L, Pahlman S (2007). Neuroblastoma as an experimental model for neuronal differentiation and hypoxia-induced tumor cell dedifferentiation. Seminars in cancer biology.

[6] Brodeur GM (2003). Neuroblastoma: biological insights into a clinical enigma. Nature reviews Cancer.

[7] Reynolds CP (2000). Differentiating agents in pediatric malignancies: retinoids in neuroblastoma. Current oncology reports.

[8] Mao L, Ding J, Zha Y, Yang L, McCarthy BA, King W (2011). HOXC9 links cell-cycle exit and neuronal differentiation and is a prognostic marker in neuroblastoma. Cancer Res.

[9] Janardhanan R, Banik NL, Ray SK (2009). N-Myc down regulation induced differentiation, early cell cycle exit, and apoptosis in human malignant neuroblastoma cells having wild type or mutant p53. Biochemical pharmacology.

[10] Guarnieri S, Pilla R, Morabito C, Sacchetti S, Mancinelli R, Fano G (2009). Extracellular guanosine and GTP promote expression of differentiation markers and induce S-phase cell-cycle arrest in human SH-SY5Y neuroblastoma cells. Int J Dev Neurosci.

[11] Georgopoulou N, Hurel C, Politis PK, Gaitanou M, Matsas R, Thomaidou D (2006). BM88 is a dual function molecule inducing cell cycle exit and neuronal differentiation of neuroblastoma cells via cyclin D1 down-regulation and retinoblastoma protein hypophosphorylation. The Journal of biological chemistry.

[12] Liu Y, Encinas M, Comella JX, Aldea M, Gallego C (2004). Basic helix-loop-helix proteins bind to TrkB and p21(Cip1) promoters linking differentiation and cell cycle arrest in neuroblastoma cells. Molecular and cellular biology.

[13] Wainwright LJ, Lasorella A, Iavarone A (2001). Distinct mechanisms of cell cycle arrest control the decision between differentiation and senescence in human neuroblastoma cells. Proceedings of the National Academy of Sciences of the United States of America.

[14] Dai MS, Mantel CR, Xia ZB, Broxmeyer HE, Lu L (2000). An expansion phase precedes terminal erythroid differentiation of hematopoietic progenitor cells from cord blood in vitro and is associated with up-regulation of cyclin E and cyclin-dependent kinase 2. Blood.

[15] Di Cunto F, Topley G, Calautti E, Hsiao J, Ong L, Seth PK (1998). Inhibitory function of p21Cip1/WAF1 in differentiation of primary mouse keratinocytes independent of cell cycle control. Science.

[16] Rots NY, Iavarone A, Bromleigh V, Freedman LP (1999). Induced differentiation of U937 cells by 1,25-dihydroxyvitamin D3 involves cell cycle arrest in G1 that is preceded by a transient proliferative burst and an increase in cyclin expression. Blood.

[17] McClellan KA, Slack RS (2006). Novel functions for cell cycle genes in nervous system development. Cell cycle.

[18] Cesi V, Tanno B, Vitali R, Mancini C, Giuffrida ML, Calabretta B (2002). Cyclin D1-dependent regulation of B-myb activity in early stages of neuroblastoma differentiation. Cell death and differentiation.

[19] Sumrejkanchanakij P, Eto K, Ikeda MA (2006). Cytoplasmic sequestration of cyclin D1 associated with cell cycle withdrawal of neuroblastoma cells. Biochemical and biophysical research communications.

[20] Zhao Z, Ma X, Hsiao TH, Lin G, Kosti A, Yu X (2014). A high-content morphological screen identifies novel microRNAs that regulate neuroblastoma cell differentiation. Oncotarget.

[21] Molenaar JJ, Ebus ME, Koster J, van Sluis P, van Noesel CJ, Versteeg R (2008). Cyclin D1 and CDK4 activity contribute to the undifferentiated phenotype in neuroblastoma. Cancer Res.

[22] Heiskanen MA, Bittner ML, Chen Y, Khan J, Adler KE, Trent JM (2000). Detection of gene amplification by genomic hybridization to cDNA microarrays. Cancer Res.

[23] Su WT, Alaminos M, Mora J, Cheung NK, La Quaglia MP, Gerald WL (2004). Positional gene expression analysis identifies 12q overexpression and amplification in a subset of neuroblastomas. Cancer Genet Cytogenet.

[24] Bayraktar S, Rocha Lima CM (2012). Emerging cell-cycle inhibitors for pancreatic cancer therapy. Expert opinion on emerging drugs.

[25] Johansson M, Persson JL (2008). Cancer therapy: targeting cell cycle regulators. Anti-cancer agents in medicinal chemistry.

[26] de Carcer G, Perez de Castro I, Malumbres M (2007). Targeting cell cycle kinases for cancer therapy. Current medicinal chemistry.

[27] Zhao Z (2014). MX, Hsiao T.H, Lin G, Kosti A, Yu X, Suresh U, Chen Y, Tomlinson G.E, Pertsemlidis A, Du L. A high-content morphological screen identifies novel microRNAs that regulate neuroblastoma cell differentation. Oncotarget.

[28] Zhao Z, Partridge V, Sousares M, Shelton SD, Holland CL, Pertsemlidis A (2018). microRNA-2110 functions as an onco-suppressor in neuroblastoma by directly targeting Tsukushi. PLoS One.

[29] Strieder V, Lutz W (2003). E2F proteins regulate MYCN expression in neuroblastomas. J Biol Chem.

[30] Feng L, Barnhart JR, Seeger RC, Wu L, Keshelava N, Huang SH (2008). Cdc6 knockdown inhibits human neuroblastoma cell proliferation. Mol Cell Biochem.

[31] Mao L (2011). DJ, Zha Y, McCarthy B.A, King W, Cui H, Ding H.F. HOXC9 links cell-cycle exit and neuronal differentiation and is a prognostic marker in neuroblastoma. Cancer Research.

[32] Cheung YT, Lau WK, Yu MS, Lai CS, Yeung SC, So KF (2009). Effects of all-trans-retinoic acid on human SH-SY5Y neuroblastoma as in vitro model in neurotoxicity research. Neurotoxicology.

[33] Foley NH, Bray I, Watters KM, Das S, Bryan K, Bernas T (2011). MicroRNAs 10a and 10b are potent inducers of neuroblastoma cell differentiation through targeting of nuclear receptor corepressor 2. Cell Death Differ.

[34] Takenobu H, Shimozato O, Nakamura T, Ochiai H, Yamaguchi Y, Ohira M (2011). CD133 suppresses neuroblastoma cell differentiation via signal pathway modification. Oncogene.

[35] Huang TC, Chang HY, Chen CY, Wu PY, Lee H, Liao YF (2011). Silencing of miR-124 induces neuroblastoma SK-N-SH cell differentiation, cell cycle arrest and apoptosis through promoting AHR. FEBS Lett.

[36] Zhao Z, Shelton SD, Oviedo A, Baker AL, Bryant CP, Omidvarnia S (2020). The PLAGL2/MYCN/miR-506-3p interplay regulates neuroblastoma cell fate and associates with neuroblastoma progression. J Exp Clin Cancer Res.

[37] Bayeva N, Coll E, Piskareva O (2021). Differentiating Neuroblastoma: A Systematic Review of the Retinoic Acid, Its Derivatives, and Synergistic Interactions. J Pers Med.

[38] Westermark UK, Wilhelm M, Frenzel A, Henriksson MA (2011). The MYCN oncogene and differentiation in neuroblastoma. Semin Cancer Biol.

[39] Zhao Z, Ma X, Shelton SD, Sung DC, Li M, Hernandez D (2016). A combined gene expression and functional study reveals the crosstalk between N-Myc and differentiation-inducing microRNAs in neuroblastoma cells. Oncotarget.

[40] Rickman DS, Schulte JH, Eilers M (2018). The Expanding World of N-MYC-Driven Tumors. Cancer Discov.

[41] Berumen J, Espinosa AM, Medina I (2014). Targeting CDKN3 in cervical cancer. Expert Opin Ther Targets.

[42] Campbell GJ, Hands EL, Van de Pette M (2020). The Role of CDKs and CDKIs in Murine Development. Int J Mol Sci.

[43] Besson A (2008). DSF, Roberts J.M. CDK inhibitors: cell cycle regualtors and beyond. Developmental Cell.

[44] Qiao L, Paul P, Lee S, Qiao J, Wang Y, Chung DH (2013). Differential regulation of cyclin-dependent kinase inhibitors in neuroblastoma cells. Biochem Biophys Res Commun.

[45] Kranenburg O (1995). SV, Van der Eb A.J, Zantema A. Inhibition of cyclin-dependent kinase activity triggers neuronal differentiation of mouse neuroblastoma cells. Journal of Cell Biology.

[46] Li T, Xue H, Guo Y, Guo K (2014). CDKN3 is an independent prognostic factor and promotes ovarian carcinoma cell proliferation in ovarian cancer. Oncol Rep.

[47] Li Y (2017). JS, Fu L, Jiang T, Wu D, Meng F.D. Knockdown of Cyclin-dependent Kinase Inhibitor 3 Inhibits Proliferation and Invasion in Human Gastic Cancer Cells. Oncology Reseach Featuring Preclinical and Clinical Cancer Therapeutics.

[48] Abdel-Tawab MS, Fouad H, Othman AM, Eid RA, Mohammed MA, Hassan A (2022). Evaluation of gene expression of PLEKHS1, AADAC, and CDKN3 as novel genomic markers in gastric carcinoma. PLoS One.

[49] Yu H, Yao J, Du M, Ye J, He X, Yin L (2020). CDKN3 promotes cell proliferation, invasion and migration by activating the AKT signaling pathway in esophageal squamous cell carcinoma. Oncol Lett.

[50] Nalepa G, Barnholtz-Sloan J, Enzor R, Dey D, He Y, Gehlhausen JR (2013). The tumor suppressor CDKN3 controls mitosis. J Cell Biol.

[51] Niculescu MD, Yamamuro Y, Zeisel SH (2004). Choline availability modulates human neuroblastoma cell proliferation and alters the methylation of the promoter region of the cyclin-dependent kinase inhibitor 3 gene. J Neurochem.

[52] Gao LM, Zheng Y, Wang P, Zheng L, Zhang WL, Di Y (2019). Tumor-suppressive effects of microRNA-181d-5p on non-small-cell lung cancer through the CDKN3-mediated Akt signaling pathway in vivo and in vitro. Am J Physiol Lung Cell Mol Physiol.

[53] Zhang M, Wang X, Yao J, Qiu Z (2019). Long non-coding RNA NEAT1 inhibits oxidative stress-induced vascular endothelial cell injury by activating the miR-181d-5p/CDKN3 axis. Artif Cells Nanomed Biotechnol.

[54] Liu D, Zhang J, Wu Y, Shi G, Yuan H, Lu Z (2018). YY1 suppresses proliferation and migration of pancreatic ductal adenocarcinoma by regulating the CDKN3/MdM2/P53/P21 signaling pathway. Int J Cancer.

[55] Schleiermacher G, Rubie H, Hartmann O, Bergeron C, Chastagner P, Mechinaud F (2003). Treatment of stage 4s neuroblastoma-report of 10 years' experience of the French Society of Paediatric Oncology (SFOP). Br J Cancer.

[56] Kawano A, Hazard FK, Chiu B, Naranjo A, LaBarre B, London WB (2021). Stage 4S Neuroblastoma: Molecular, Histologic, and Immunohistochemical Characteristics and Presence of 2 Distinct Patterns of MYCN Protein Overexpression-A Report From the Children's Oncology Group. Am J Surg Pathol.

